# Regulatory Roles of Non-Coding RNAs in Cancer

**DOI:** 10.3390/cells12091298

**Published:** 2023-05-02

**Authors:** Macrina B. Silva-Cázares, Carlos Pérez-Plasencia, César López-Camarillo

**Affiliations:** 1 Coordinación Académica Región Altiplano, Universidad Autónoma de San Luis Potosí, San Luis Potosí 78760, Mexico; macrina.silva@uaslp.mx; 2 Laboratorio de Genómica, Instituto Nacional de Cancerologia, Mexico City 14080, Mexico; carlos.pplas@gmail.com; 3 Posgrado en Ciencias Genómicas, Universidad Autónoma de la Ciudad de México, Mexico City 03100, Mexico

For several decades, scientific research in cancer biology has focused mainly on the involvement of protein-coding genes. Recently, however, a whole class of molecules known as non-coding RNA (ncRNA) have been discovered to be crucial in regulating cell function. Intensive study of ncRNA biology has revealed that these molecules form a diverse and abundant group of RNAs that include both tumor-promoting and cancer-preventing molecules [1]. 

The development of RNA sequencing and computational methods significantly enhanced the study of non-coding RNA (ncRNA). Once considered insignificant products of complex transcription, ncRNAs were later found to play important roles in various aspects of biological function. They are key players in gene regulatory networks along side other biomolecules (DNA, RNA or protein). Innovative sequencing techniques and computational tools must be fully exploited to better investigate ncRNA-mediated regulation [2] 

The fact that a single ncRNA can influence the expression of various downstream gene targets and related pathways has led to an investigation of ncRNAs as drug targets for cancer therapy. This highlights the importance of collecting relevant data and developing new approaches to overcome the challenges of their delivery and minimize the side effects of human cancer [3]. 

This Special Issue, entitled “Regulatory roles of non-coding RNAs in cancer”, presents the recent advances in the understanding of the role of regulatory ncRNAs such as microRNAs, lncRNAs, ceRNAs and circular RNAs in the mechanisms of cancer development and progression. The publication highlights research that focuses on characterizing new ncRNAs as potential biomarkers and describing ncRNA-based therapies that target genes that affect cancer. Szczepanek et al. summarizes the role of microRNAs in cancer therapy and presents therapeutic concepts for these molecules. Clinical studies have already shown the possibility of supporting cancer treatment with microRNA molecules [4]. 

A systematic review by Calorden et al. revealed the presence of circulating microRNAs in the serum of endometrial cancer patients and their association with the clinical and prognostic features of endometrial cancer. The review revealed significant expression differences in EC patients compared to healthy individuals. However, on another hand, more studies should be conducted to better understand the function and circulation of these miRs [5]. The relevance of microRNA (miRNA) in the development and spread of colon cancer has, furthermore, been highlighted. Williams et al. created a metric based on the miRNA–target gene interactions previously found in colon cancer; their method outperforms existing techniques, and the essential genes identified by their approach are involved in NOTCH3 signaling and common metabolic pathways critical to colon cancer progression [6].

Wu et al. investigated the role of a long noncoding RNA (lncRNA), known as LOC550643, in colorectal cancer (CRC) cell growth and metastasis. Their study found that the LOC550643-miR-29b-2-5p axis is involved in these processes, but the exact mechanism remains unclear. The study suggests that this axis may be a molecular biomarker for cancer diagnosis and a potential therapeutic target for CRC [7]. In their work, Maldonado et al. reviewed the current understanding of the extent to which noncoding RNAs (lncRNAs) are involved in the hypoxic response of cancer cells and how they affect cancer hallmarks. They highlight the central role of lncRNAs in the regulation of the upstream and downstream signaling pathways of hypoxia, including the HIF-1α pathway, and describe how they can influence cancer cell proliferation, angiogenesis, invasion and metastasis. In addition, they discuss the potential of hypoxia-related lncRNAs as diagnostic and prognostic biomarkers and their potentials as therapeutic targets in cancer therapy [8]. Pellegrino et al. discovered an epigenetic mechanism leading to a long intergenic noncoding RNA 152 (LINC00152) expression in human hepatocellular carcinoma (HCC). Their study identified a possible competitive endogenous RNA (ceRNA) network [0] in which LINC00152 exerts oncogenic functions via fungal miRNAs by causing changes in target gene expression. They identified KLC2 as a key mediator contributing to the protumorigenic effects of LINC00152 overexpression in HCC cells. Ref. [9] Interesting observations have also been reported by Arancio et al. regarding the expression of repetitive sequences in breast cancer samples. It is noteworthy that an increase in satellite sequences is associated with certain breast cancer histotypes. This information could potentially be helpful for the diagnosis of breast cancer and identification of subtypes. In addition, the observation of an interruption of the transcription of endogenous retroviruses and their LTR sequences indicates the possible role of these sequences in the development and progression of breast cancer. Further research in this area may provide valuable insights into the molecular mechanisms underlying breast cancer [10].

Circular RNAs (circRNAs) are a relatively new group of noncoding RNAs that have been found to play important roles in various biological processes, including cancer. Szczepaniak et al. provides an overview of the current understanding of the role of circRNAs in cancer, with a particular focus on cancer stem cells. They summarize the various mechanisms by which circRNAs can influence cancer progression and discuss the latest tools available with which circRNAs can be studied. The review highlights the potential of circRNAs as diagnostic and prognostic markers and targets for cancer therapy [11]. This is an interesting finding by Nuñez-Olvera et. al. The use of co-expressed lncRNAs/mRNAs pairs as preclinical tools to identify biomarkers related to response to endocrine therapy and to understand the biological behavior of cancer cells in 3D microenvironments could have significant implications in the development of personalized cancer treatment. It is important to note that further studies are needed to confirm the results and assess their clinical utility [12].
